# Study protocol: a core outcome set for perinatal interventions for congenital diaphragmatic hernia

**DOI:** 10.1186/s13063-021-05120-z

**Published:** 2021-02-23

**Authors:** Simen Vergote, Felix De Bie, Jan Bosteels, Holly Hedrick, James Duffy, Beverley Power, Alexandra Benachi, Paolo De Coppi, Caraciolo Fernandes, Kevin Lally, Irwin Reiss, Jan Deprest

**Affiliations:** 1 Department of Development and Regeneration cluster Woman and Child, Biomedical Sciences, KU Leuven, Herestraat 49 - Box 805, B-3000 Leuven, Belgium; 2 Department of Surgery, Children’s Hospital of Philadelphia, Philadelphia, PA USA; 3 Institute of Women’s Health, University College of London, London, UK; 4 Nuffield Department of Primary Care Health Sciences, University of Oxford, Oxford, UK; 5 CDH UK, The Denes, Norfolk, UK; 6 Service de Gynécologie-Obstétrique, Hôpital Antoine Béclère, AP-HP, Clamart, France; 7 Department of Specialist Neonatal and Pediatric Surgery, Great Ormond Street Hospital for Children, Great Ormond Street, London, UK; 8 Stem Cells and Regenerative Medicine Section, Institute of Child Health, University College London, London, UK; 9 Texas Children’s Fetal Center, Texas Children’s Hospital and Department of Pediatrics - Newborn Section, Baylor College of Medicine, Houston, TX USA; 10 Department of Pediatric Surgery, McGovern Medical School at UT Health and Children’s Memorial Hermann Hospital, Houston, TX USA; 11 Department of Pediatrics, Division of Neonatology, Erasmus MC, University Medical Center Rotterdam, Rotterdam, Netherlands

**Keywords:** Congenital diaphragmatic hernia, Perinatal intervention, Core outcome set, Delphi method

## Abstract

**Background:**

Congenital diaphragmatic hernia (CDH) is, depending of the severity, a birth defect associated with significant mortality and morbidity. Prenatal screening by ultrasound may detect this condition and comprehensive assessment of severity is possible, allowing for in utero referral to an experienced centre for planned delivery. In an effort to improve outcomes, prenatal interventions to stimulate lung development were proposed. Along the same lines, new postnatal management strategies are being developed. In order to enable proper comparison of novel perinatal interventions as well as outcomes, a set of uniform and relevant outcome measures is required. Core outcome sets (COS) are agreed, clearly defined sets of outcomes to be measured in a standardised manner and reported consistently. Herein we aim to describe the methodology we will use to define a COS for perinatal and neonatal outcomes of foetuses and newborns with congenital diaphragmatic hernia and to draft a dissemination and implementation plan.

**Methods:**

We will use the methodology described in the Core Outcome Measures in Effectiveness Trials (COMET) Initiative Handbook. An international steering group will be created to guide the development of the COS. We are systematically reviewing the literature to identify all potential relevant pre- and neonatal outcomes previously used in studies on perinatal interventions for CDH. We will build a consensus on these core outcomes in a stakeholder group using the Delphi method. After completion, a stakeholder meeting will decide on a final COS, using a modified Nominal Group Technique. Thereafter, we will review potential definitions and measurements of these outcomes, and again a consensus meeting will be organised, to finalise the COS before dissemination.

**Discussion:**

We have started a procedure to develop a COS for studies on perinatal interventions for congenital diaphragmatic hernia, with the purpose of improving the quality of research, guide clinical practice and improve patient care and eventual use in future clinical trials, systematic reviews and clinical practice guidelines.

**Trial registration:**

We prospectively registered this study in the International Prospective Register of Systematic Reviews (PROSPERO) (registration number: CRD42019124399) and The Core Outcome Measures in Effectiveness Trials (COMET) Initiative (registration number:1296).

## Background

Core outcome sets (COS) are agreed, clearly defined minimum sets of outcomes that should be measured in a standardised manner and reported consistently [1]. The use of COS leads to higher-quality trials and facilitates comparison, contrasting and combination of trial results, hence reducing waste of time and resources in research. Over the last years, a number of core outcome sets have been successfully developed and implemented for foetal pathologies including twin-to-twin transfusion syndrome and selective foetal growth restriction [2, 3].

Congenital diaphragmatic hernia (CDH) is a congenital defect with a prevalence at birth of approximately 2.3 in 10,000 [4]. This defect involves partial or complete absence of the diaphragm, leading to herniation of the viscera into the thoracic cavity, impairing prenatal lung development [5]. At birth, this manifests as respiratory insufficiency and pulmonary hypertension, which leads to neonatal death in approximately 30% of cases despite neonatal care in specialised high-volume tertiary centres with standardised protocols [6]. Survivors may have serious morbidities, mostly cardio-respiratory in nature but also feeding problems, reflux, growth and orthopaedic problems.

Advances in prenatal ultrasound enable CDH to be diagnosed prenatally in 68% [7]. This allows for assessment of severity and in utero referral for planned delivery [8]. Prenatal surgical interventions have been developed to improve lung size and consequently improve lung function and hence neonatal outcome [9]. One such intervention is fetoscopic endoluminal tracheal occlusion (FETO) which is currently being evaluated in a multicentre randomised controlled trial (www.TOTALtrial.eu) and may improve survival in isolated CDH with severe pulmonary hypoplasia, as compared to the standard perinatal management [10, 11]. Besides novel surgical techniques, medical interventions are evaluated aiming to further improve the prognosis [12, 13]. Unfortunately, investigators report a variety of outcomes, making comparison or combination of data from individual studies very challenging and limiting the potential of research to guide clinical practice. We therefore believe that creation of a COS is urgently needed to improve the quality and efficiency of research in CDH and to increase the effectiveness of both the dissemination and implementation of study findings. Herein we describe the methodology we propose to use to define a COS for perinatal and neonatal outcomes in CDH. We also present a dissemination and implementation plan for the proposed COS.

## Methods

**Fig. 1:**
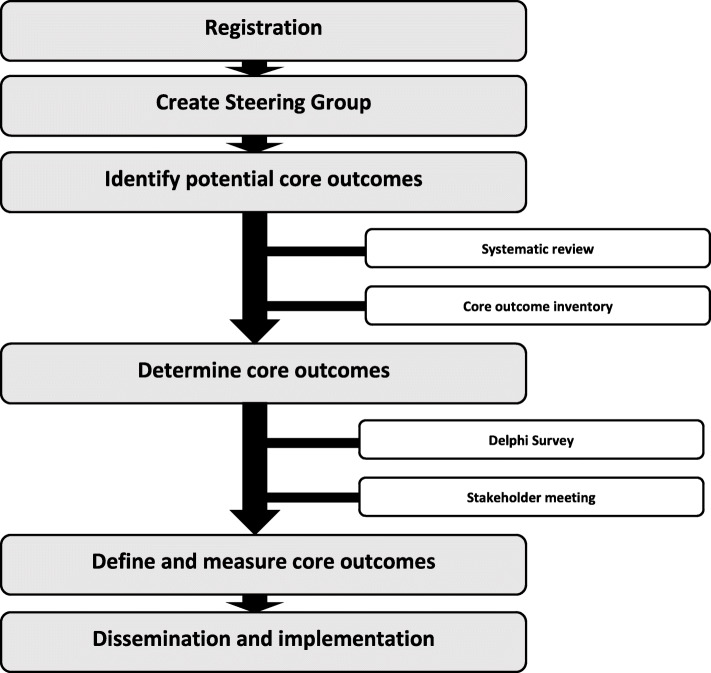
Stages of developing a core outcome set for CDH

We will use the methodology described in the Core Outcome Measures in Effectiveness Trials (COMET) Initiative Handbook, which we have previously used for the development of other COS in perinatal research, e.g. as for twin-to-twin transfusion syndrome, pre-eclampsia and selective foetal growth restriction [1, 14–16]. The study protocol as outlined below meets the recommendations described in the “Standard Protocol Items: Recommendations for Interventional Trials” (SPIRIT) document [17]. The stages of the development of the COS are presented in Fig. 1.

### Registration

We have registered our study in the International Prospective Register of Systematic Reviews (PROSPERO) (registration number: CRD42019124399) and The Core Outcome Measures in Effectiveness Trials (COMET) Initiative (registration number: 1296). We will use the PRISMA Statement guidelines for Reporting Systematic Reviews and Meta-Analyses of Studies that Evaluate Health Care Interventions for our systematic review [18].

### Steering group

To guide the development of the COS, we will form an international steering group (ISG) constituted of at least two members with expertise in CDH of each of the following groups: maternal-foetal medicine specialists, researchers, methodologists, paediatric surgeons, neonatologists and patient organisation-representatives. The ISG will critically appraise the methodology, agree on the composition of the stakeholder group, discuss and approve the core outcome inventory, pilot-test a first round of the Delphi survey and join the stakeholder group in completing the Delphi survey. Patient organisation-representatives will also be involved in the wording of the outcomes and advertising the survey to patients. We will establish a management group within the ISG for the day-to-day management of the progress of the study.

### Scope of the core outcome set

CDH is defined by Orphanet as a defect of the diaphragm that allows passage of abdominal viscera into the thorax, leading to pulmonary hypoplasia, causing respiratory insufficiency and pulmonary hypertension with high mortality (ORPHA:2140). The COS will not be limited by the type, side or severity of CDH. The COS will apply to peri- and neonatal outcomes in studies evaluating perinatal interventions for CDH. We defined “perinatal interventions” as all prenatal interventions and interventions at the time of delivery. All perinatal therapeutic interventions will be considered, regardless of type, mode of administration or the gestation at which provided. The “neonatal period” was defined as 28 days after birth, as stated by the World Health Organization. We do not aim to reach consensus on the standardisation of other aspects of study design or the definition of CDH.

### Identifying potential core outcomes

We will conduct a systematic review to collect all reported outcomes in the literature. When designing our search strategy, we will carefully consider our methodological decisions taking into account the experiences of other core outcome set developers [19, 20]. We will search the Cochrane Central Register of Controlled Trials (CENTRAL), the EMBASE and the MEDLINE databases for trials and observational studies reporting on peri- and neonatal outcomes for perinatal interventions for CDH. We will create a search strategy consisting of relevant MeSH terms and free-text words using key words for CDH and all possible interventions.Randomised controlled trials and observational studies will be included. Reviews, case reports (< 3 cases), incomplete reports, book chapters, conference abstracts, letters to the editors and comments will be excluded. No language limits will be applied. Studies identified by the search will be screened on title and abstract by two reviewers independently (SV and FDB). If inclusion cannot be agreed upon based on the abstract, or if no abstract is available, we will search the full text of the article. All full-text articles will be reviewed by two researchers (SV and FDB) for possible inclusion. If consensus cannot be reached, arbitration will be sought by a third reviewer (JB).

Using a standardised data-extraction file (Microsoft Excel, Microsoft Corp, Redmond, WA, USA), we aim to collect the following information: year of study, journal, study design, sample size, participant characteristics, side of the defect, type of intervention(s), funding source and all reported pre- and neonatal outcomes. All identified outcomes will be entered into an outcome inventory and organised into the following categories: foetal outcomes, obstetric outcomes, neonatal outcomes, maternal outcomes, prenatal intervention outcomes and postnatal intervention outcomes. Such thematic organisation has been successfully used in other COS development studies [2, 3, 21]. All outcomes will be reviewed and discussed by the ISG with particular emphasis on reducing duplication of outcomes caused by varying terminologies and grouping very similar outcomes together to make the final outcome inventory clear and succinct. Following the agreement, the inventory will be entered into the modified Delphi survey using Delphi survey software (DelphiManager, University of Liverpool, Liverpool, United Kingdom). The wording of the outcomes will be agreed upon after consultation of the patient organisation representatives.

### Determining core outcomes

The core outcomes will be determined using a modified Delphi method, an established tool for achieving consensus by gathering data from respondents with expert knowledge of the subject under study. The Delphi method allows consensus-building by using a series of questionnaires to extract opinions from participants [16, 22].

The ISG will suggest potential participants to be invited for filling out the questionnaires. Additionally, we aim to identify potential participants by advertising the study by newsletters and/or online media of national and international patient organisations, including CDH UK, PlatformCHD, the European Reference Network ERNICA, and other relevant professional organisations. There is no robust method for calculating the required sample size but typically groups have included 13–222 participants [22]. We aim to include at least 18 members per stakeholder category, i.e. patient representatives, researchers and health professionals with a known expertise in CDH [14, 16]. Potential participants can register their interest online and membership of the stakeholder group will be determined by the study management group, based on their profile. If there are insufficient members, the steering committee will re-advertise for membership.

### Delphi survey

Next, we will send instructions written in plain language by email to the stakeholders, inviting them to fill out the questionnaires, further referred to as the “Delphi survey”. This survey includes demographic details, as well as information relating to stakeholders’ experiences with or expertise on CDH. The survey will be semi-anonymous: all participants of the Delphi-survey will be allocated a unique identifier which does not include information that can identify the participant (name, address or telephone number). Only members of the study management group can link the unique identifier to the contact information of the participants in order to contact them to complete the survey or to request feedback on their reasons for not doing so.

A Delphi survey consists of at least two rounds [1]. In the first round, participants are invited to score the relevance of given outcomes on a generic nine-point Likert scale. This scale was devised by the Grading of Recommendations Assessment, Development and Evaluation (GRADE) working group to facilitate the ranking of outcomes according to their importance, and has been adopted widely by core outcome set developers [23]. Stakeholders will also have the opportunity to suggest additional outcomes. These outcomes will be reviewed and where needed standardised by the study management group; relevant outcomes are incorporated in the next Delphi round. The latter and the former, initial outcomes are carried forward into the second round. For each outcome, the frequency distribution over the nine-point Likert scale will be tabulated for each stakeholder category. Upon disclosure of the scores per group, individual stakeholders will be given the chance to rescore outcomes. Eventually, we will use the following standardised criteria to determine whether a given core outcome should be included in the final COS or discarded [1, 14]:

1. *Consensus in* (classify as a core outcome): Over 70% of participants in all stakeholder groups score the outcome ‘critical for decision making’ (score seven to nine) and less than 15% of participants in all stakeholder group score the outcome ‘of limited importance for decision making’ (score one to three).
2. *Consensus out* (do not classify as a core outcome): Over 70% of participants in all stakeholder groups score the outcome ‘of limited importance for decision making’ (score one to three) and less than 15% of participants in all stakeholder groups score the outcome ‘critical for decision making’ (score seven to nine).
3. *No consensus* (do not classify as a core outcome): Anything else in between the criteria for 1 and 2.

The results of the second round will be reviewed by the ISG to decide if another round is indicated e.g. due to a lack of consensus. Each round will close after 4 weeks but this timeframe will be extended in case of insufficient response. Results will be analysed using SPSS (v.26; IBM Software, Inc., Armonk, NY, USA) and displayed as median with interquartile range in frequency tables.

### Stakeholder meeting

All stakeholders who complete the survey will be invited to a face-to-face meeting. Those unable to attend the meeting will be allowed to attend by teleconference. The aim of this stakeholder meeting is to discuss all outcomes for which no consensus was reached. We will use a modified Nominal Group Technique to achieve unbiased and participatory consensus formation. This structured discussion technique allows all opinions to be considered from the start, encourages equal participation and allows the identification of divergence in opinion between different groups in a safe manner [24]. This technique has been successfully used in the development of several COS, e.g. in perinatology [2, 3]. Outcomes on which no consensus can be reached will be rejected. The result will be a final COS for perinatal interventions for CDH.

### Measuring core outcomes

In the next stage, we will define all included core outcomes and indicate how they are to be measured. Potential definitions and measurement instruments will be inventoried across formal definition development initiatives, national and international guidelines, Cochrane systematic reviews and published studies. Potential definitions will be discussed in a consensus development meeting including health professionals, researchers and patient representatives. The objective of this meeting will be to agree on definitions for individual core outcomes. Potential measurement instruments will be qualitatively assessed using the Core Outcome Measures in Effectiveness Trials (COMET) and the Consensus-Based Standards for the Selection of Health Measurement Instruments (COSMIN) Initiative quality assessment framework [25].

### Dissemination and implementation

The final COS will be disseminated by the steering group to be used as widely and as efficiently as possible. We aim to publish the set in a relevant journal and present it whenever possible to our peers in meetings. Relevant societies, including the International Fetal Medicine and Surgery Society, the International Society on Prenatal Diagnosis, the European Reference Network ERNICA and other professional organisations, as well as patient organisations, such as CDH UK and PlatformCHD, will be determined by the steering group and contacted to assist in disseminating our findings to patient representatives and clinicians worldwide.

## Discussion

Once a COS has been defined, we hope that its implementation in future clinical studies, prospective databases, systematic reviews and clinical guidelines will advance the reach and relevance of research on perinatal interventions for CDH, inform clinical practice, enhance patient care and improve maternal and offspring outcomes. Ultimately, we aim to improve effective clinical care and patients’ experience.

### Improving clinical trial outcome selection

The Standard Protocol Items: Recommendations for Interventional Trials (SPIRIT) Statement, supported by funders of health research, such as the National Institute of Health Research (NIHR), strongly recommends the use of COS [17]. The use of standard core outcome sets enhances the comparability of clinical trials and facilitates the conduct of using individual patient data meta-analysis (IPD). A recently published core outcome set for preterm birth has been successfully implemented across 12 randomised trials being undertaken in six different countries [26, 27].

### Improving clinical trial reporting and evidence synthesis

The Core Outcomes in Women’s and Newborn’s Health (CROWN) Initiative, supported by over 80 specialty journals, including the Cochrane Pregnancy and Childbirth Group, intends to implement COS [28, 29]. Participating journals expect authors to report study results for the core outcomes and draw their conclusions based on these, rather than on non-core or surrogate outcomes. If core outcomes have not been collected, authors are typically asked to report their failure not to do so, justify it, and comment on the implications for their findings. This eventually enhances the probability of developing robust clinical guidelines [28].

### Improving clinical practice guidelines

The National Institute for Health and Care Excellence (NICE) supports the use of COS during evidence scoping and synthesis [17, 28]. As this activity forms the basis of updating guideline recommendations, COS can have a direct impact in influencing clinical practice and patient outcomes.

### Developing infrastructure to support international collaboration

Developing a COS will establish an international network of key stakeholders in CDH, including healthcare professionals, researchers, and patient representatives with experience of contributing to a collaborative online study. This infrastructure could be leveraged in other settings, for example selecting research priorities and clinical practice guideline development.

## Conclusion

Core outcome sets are clearly defined minimum sets of outcomes that should be reported consistently. Guided by an international steering group, we aim to create a COS relevant to perinatal interventions for CDH, to improve the quality of research and eventually influence clinical practice.

### Trial status

At the time of manuscript submission, we are analysing the results of the systematic review and constructing the Delphi survey.

## Data Availability

Not applicable.

## Abbreviations

CDH: Congenital Diaphragmatic Hernia
CENTRAL: Cochrane Central Register of Controlled Trials
COMET: Core Outcome Measures in Effectiveness Trials Initiative
CROWN: Core Outcomes in Women’s and Newborn’s Health
GRADE: Grading of Recommendations Assessment, Development and Evaluation
MeSH: Medical Subject Headings
COSMIN: Consensus-Based Standards for the Selection of Health Measurement Instruments
NICE: National Institute for Health and Care Excellence
NIHR: National Institute of Health Research
PRISMA: Preferred Reporting Items for Systematic Reviews and Meta-analyses
PROSPERO: International Prospective Register of Systematic Reviews
SPIRIT: Standard Protocol Items: Recommendations for Interventional Trials

## References

[1] Williamson PR, Altman DG, Bagley H, Barnes KL, Blazeby JM, Brookes ST (2017). The COMET Handbook: version 1.0. Trials.

[2] Perry H, Duffy JMN, Reed K, Baschat A, Deprest J, Hecher K (2019). Core outcome set for research studies evaluating treatments for twin-twin transfusion syndrome. Ultrasound Obstet Gynecol.

[3] Townsend R, Duffy JMN, Sileo F, Perry H, Ganzevoort W, Reed K, et al. Core outcome set for studies investigating management of selective fetal growth restriction in twins. Ultrasound Obstet Gynecol. 2020;55(5):652–60. 10.1002/uog.20388 31273879

[4] McGivern MR, Best KE, Rankin J, Wellesley D, Greenlees R, Addor MC (2015). Epidemiology of congenital diaphragmatic hernia in Europe: a register-based study. Arch Dis Child Fetal Neonatal Ed.

[5] Ameis D, Khoshgoo N, Keijzer R (2017). Abnormal lung development in congenital diaphragmatic hernia. Semin Pediatr Surg.

[6] Harting MT, Lally KP (2014). The congenital diaphragmatic hernia study group registry update. Semin Fetal Neonatal Med.

[7] Burgos CM, Frenckner B, Luco M, Harting MT, Lally PA, Lally KP (2019). Prenatally versus postnatally diagnosed congenital diaphragmatic hernia - side, stage, and outcome. J Pediatr Surg.

[8] Russo FM, Cordier AG, De Catte L, Saada J, Benachi A, Deprest J (2018). Proposal for standardized prenatal ultrasound assessment of the fetus with congenital diaphragmatic hernia by the European reference network on rare inherited and congenital anomalies (ERNICA). Prenat Diagn.

[9] Grivell RM, Andersen C, Dodd JM (2015). Prenatal interventions for congenital diaphragmatic hernia for improving outcomes. Cochrane Database Syst Rev.

[10] Al-Maary J, Eastwood MP, Russo FM, Deprest JA, Keijzer R (2016). Fetal tracheal occlusion for severe pulmonary hypoplasia in isolated congenital diaphragmatic hernia: a systematic review and meta-analysis of survival. Ann Surg.

[11] Deprest J, Gratacos E, Nicolaides KH, Group FT (2004). Fetoscopic tracheal occlusion (FETO) for severe congenital diaphragmatic hernia: evolution of a technique and preliminary results. Ultrasound Obstet Gynecol.

[12] Russo FM, Toelen J, Eastwood MP, Jimenez J, Miyague AH, Vande Velde G (2016). Transplacental sildenafil rescues lung abnormalities in the rabbit model of diaphragmatic hernia. Thorax..

[13] Russo FM, De Coppi P, Allegaert K, Toelen J, van der Veeken L, Attilakos G (2017). Current and future antenatal management of isolated congenital diaphragmatic hernia. Semin Fetal Neonatal Med.

[14] Duffy JM, van’t Hooft J, Gale C, Brown M, Grobman W, Fitzpatrick R (2016). A protocol for developing, disseminating, and implementing a core outcome set for pre-eclampsia. Pregnancy Hypertens.

[15] Khalil A, Duffy JMN, Perry H, Ganzevoort W, Reed K, Baschat AA (2019). Study protocol: developing, disseminating, and implementing a core outcome set for selective fetal growth restriction in monochorionic twin pregnancies. Trials..

[16] Khalil A, Perry H, Duffy J, Reed K, Baschat A, Deprest J (2017). Twin-twin transfusion syndrome: study protocol for developing, disseminating, and implementing a core outcome set. Trials..

[17] Chan A-W, Tetzlaff JM, Gøtzsche PC, Altman DG, Mann H, Berlin JA (2013). SPIRIT 2013 explanation and elaboration: guidance for protocols of clinical trials. BMJ.

[18] Liberati A, Altman DG, Tetzlaff J, Mulrow C, Gotzsche PC, Ioannidis JP (2009). The PRISMA statement for reporting systematic reviews and meta-analyses of studies that evaluate healthcare interventions: explanation and elaboration. BMJ..

[19] Duffy J, Hirsch M, Ziebland S, McManus R, Pre-eclampsia tICtHOi (2019). Methodological decisions influence the identification of potential core outcomes in studies related to pre-eclampsia: an analysis informing the development of recommendations for future core outcome set developers. BJOG Int J Obstet Gynaecol.

[20] Duffy J, McManus RJ. Influence of methodology upon the identification of potential core outcomes: recommendations for core outcome set developers are needed. BJOG. 2016;123(10):1599. 10.1111/1471-0528.14219 27428666

[21] Duffy J, Hirsch M, Kawsar A, Gale C, Pealing L, Plana MN (2017). Outcome reporting across randomised controlled trials evaluating therapeutic interventions for pre-eclampsia. Bjog..

[22] Sinha IP, Smyth RL, Williamson PR (2011). Using the Delphi technique to determine which outcomes to measure in clinical trials: recommendations for the future based on a systematic review of existing studies. PLoS Med.

[23] Guyatt GH, Oxman AD, Kunz R, Atkins D, Brozek J, Vist G (2011). GRADE guidelines: 2. Framing the question and deciding on important outcomes. J Clin Epidemiol.

[24] Rankin NM, McGregor D, Butow PN, White K, Phillips JL, Young JM (2016). Adapting the nominal group technique for priority setting of evidence-practice gaps in implementation science. BMC Med Res Methodol.

[25] Prinsen CA, Vohra S, Rose MR, Boers M, Tugwell P, Clarke M (2016). How to select outcome measurement instruments for outcomes included in a “Core outcome set” - a practical guideline. Trials..

[26] van’t Hooft J, Alfirevic Z, Asztalos E, Biggio J, Dugoff L, Hoffman M (2018). CROWN initiative and preterm birth prevention: researchers and editors commit to implement core outcome sets. BJOG Int J Obstet Gynaecol.

[27] van’t Hooft J, Duffy JM, Daly M, Williamson PR, Meher S, Thom E (2016). A core outcome set for evaluation of interventions to prevent preterm birth. Obstet Gynecol.

[28] Khan K (2014). The CROWN initiative: journal editors invite researchers to develop core outcomes in women's health. BJOG Int J Obstet Gynaecol.

[29] Duffy J, Rolph R, Gale C, Hirsch M, Khan K, Ziebland S (2017). Core outcome sets in women's and newborn health: a systematic review. BJOG Int J Obstet Gynaecol.

